# Trigeminocardiac Reflex Associated With Orthognathic Surgery: An Illustrative Case Report and Review of the Literature

**DOI:** 10.7759/cureus.101454

**Published:** 2026-01-13

**Authors:** Asiya Yusuf, Ashley Ferro, Oliver Vij, Thomas Mathew, Jithesh Appukutty, Vijayarajan Santhanam

**Affiliations:** 1 Oral and Maxillofacial Surgery, Cambridge University Hospitals NHS Foundation Trust, Cambridge, GBR; 2 Anesthesia, Cambridge University Hospitals NHS Foundation Trust, Cambridge, GBR

**Keywords:** le fort 1 osteotomy, orthognathic, reversible bradycardia, transient asystole, trigeminocardiac reflex trigeminal nerve

## Abstract

The trigeminocardiac reflex (TCR) is a rare brainstem reflex characterized by bradycardia and hemodynamic instability secondary to stimulation of branches of the trigeminal nerve. In maxillofacial surgery, the phenomenon has been reported in orthognathic surgery, foreign body removal, and fixation of midface fractures. Despite reasonably extensive reporting of the reflex in head and neck surgery in general, the mechanism and predisposing factors are poorly understood. The primary aim of this systematic review was to explore potential contributory factors and summarize outcomes of the TCR in patients undergoing orthognathic surgery. We additionally report a case of asystole in a 21-year-old male undergoing Le Fort I osteotomy. A systematic literature search was undertaken using MEDLINE, Embase, Web of Science, Scopus, and PubMed from inception to December 24, 2025, for articles reporting patients experiencing TCR during orthognathic surgery. Studies reporting TCR cases were identified and screened by two independent reviewers. Studies reporting TCR cases were identified and screened by two independent reviewers. Preferred Reporting Items for Systematic Reviews and Meta-Analysis (PRISMA) guidelines were followed. A total of 261 studies were initially identified, documenting 17 cases in addition to the current case. Maxillary downfracture or mobilization were the most commonly reported precipitants; however, more innocuous triggers were also identified. While cardiopulmonary resuscitation was necessary in two documented cases, all cases reported completion of the procedure with no known long-term adverse outcomes. We discuss the mechanism of the reflex in relation to the identified cases and existing literature.

## Introduction

The incidence of intraoperative bradycardia in Oral and Maxillofacial surgery was initially described by Aschner and Dagnini in 1908 and attributed to the oculocardiac reflex [1]. The phenomenon, designated as the "Aschner phenomenon," revealed that transient bradycardia was precipitated by traction on the extraocular muscles and an increase in ocular pressure, and could be elicited by applying direct pressure on the globe. A similar response was subsequently noted in non-orbital procedures, prompting recognition of the trigeminocardiac reflex (TCR). Clinically, this reflex may manifest as sudden bradycardia, hypotension, or even transient cardiac arrest, resolving when the stimulus is removed. It is now understood that the reflex is mediated via an interaction between afferent fibers of the trigeminal nerve and both the brainstem reticular formation and motor nucleus of the vagus nerve, provoking the physiological changes observed during the reflex.

Orthognathic procedures such as Le Fort osteotomies, bilateral sagittal split osteotomies (BSSO), and genioplasty involve direct mechanical stimulation, traction, or pressure on branches of the maxillary and mandibular divisions of the trigeminal nerve. These procedures involve controlled osteotomies and mobilization of the midface and mandible, often requiring substantial mechanical force, and are recognized manoeuvres for triggering the TCR [2]. Despite this, the true incidence of TCR during orthognathic surgery remains uncertain, with reported rates varying widely across the literature. Inconsistencies in terminology, diagnostic criteria, and reporting practices further complicate the interpretation and comparison of published data.

Ragno et al. reported the first published case of the TCR in orthognathic surgery in 1989, whereby a 17-year-old patient developed asystole following downfracture of the maxilla during Le Fort I osteotomy [3]. With increasing recognition of the reflex beyond the context of oral and maxillofacial Surgery, there has been increasing interest in better understanding the underlying mechanism and identifying modifiable risk factors, and it is now recognized that there are peripheral, central, and ganglion Gasseri subtypes [4]. Several factors have been suggested to influence the occurrence of TCR, including the type and intensity of surgical stimulus, depth of anesthesia, patient-related characteristics, and perioperative physiological variables [5]. However, much of the available evidence is derived from isolated case reports, small case series, or mixed craniofacial cohorts, limiting the applicability of findings specifically to orthognathic patients. As a result, the contributory factors associated with TCR in this population have not been clearly defined.

Given the elective nature of orthognathic surgery and the typically young and otherwise healthy patient population, improved understanding of perioperative risks is essential. Failure to recognize TCR intraoperatively may result in unnecessary escalation, prolonged cardiac arrest, or premature termination of surgery. The primary aim of this case report, with an accompanying review of the literature, is to evaluate reported cases of TCR in patients undergoing orthognathic surgery and to identify potential contributory factors associated with its occurrence. By synthesizing the available evidence, this review seeks to clarify current knowledge and highlight areas requiring further investigation.

## Case presentation

A healthy 21-year-old Caucasian male with a skeletal class III malocclusion was admitted electively for Le Fort I maxillary osteotomy and bilateral mandibular sagittal split osteotomy under general anesthesia. Pre-operative assessment and physical examination were unremarkable, and there was no relevant past medical history. He had no family history of cardiovascular or hematological disease.

Throughout the procedure, the patient underwent standard noninvasive anesthetic monitoring, including noninvasive blood pressure (NIBP), pulse oximetry, electrocardiography (ECG), and depth-of-anesthesia monitoring (entropy). General anesthesia was induced and maintained using total intravenous anesthesia (TIVA) with propofol and remifentanil infusions (0.2 µg/kg/min). The patient was paralyzed with an atracurium bolus prior to endotracheal intubation. A magnesium infusion (2 mmol) and clonidine boluses (105 µg) were administered to facilitate hypotension and analgesia. Additional agents administered before the episode included midazolam 2 mg, dexamethasone 6.6 mg, amoxicillin-clavulanate 1.2 g, and 200 mL of Hartmann’s solution (Table 1).

**Table 1:** Overview of pharmacologic agents used in each phase of the procedure All agents were delivered intravenously unless otherwise specified. MAP: mean arterial pressure; TIVA: total intravenous anesthesia

Phase of procedure	Pharmacologic agents
Induction and maintenance anesthesia	Propofol+remifentanil (TIVA)
Paralysis	Atracurium
MAP management	Magnesium infusion (2 mmol); clonidine bolus (105 µg)
Adjunctive agents at induction	Midazolam 2 mg; dexamethasone 6.6 mg; co-amoxiclav 1.2 g IV; Hartmann’s compound sodium lactate solution 200 mL
Local anesthesia	A 17.6 mL lidocaine hydrochloride 2%+1:80000 adrenaline via infiltration, divided between the maxillary and mandibular operative sites
During asystole	Atropine 60 µg

The surgery proceeded uneventfully with the patient positioned supine. Local anesthetic (17.6 mL of lidocaine hydrochloride 2% 1:80000 adrenaline) was administered, divided between maxillary and mandibular sites. A standard vestibular approach was used, and the Le Fort I osteotomy, including downfracture of the maxilla, was completed. No preceding changes in heart rate variability or physiological disturbance to this point were noted. During mobilization of the maxilla using Rowe’s maxillary disimpaction forceps, there was a rapid escalation into severe sinus bradycardia followed by complete ventricular asystole. An asystolic period, directly witnessed by the anesthetic and surgical team, lasted approximately 20 seconds, during which no return of QRS complexes was seen on the ECG. The operating table was immediately repositioned to allow cardiopulmonary resuscitation (CPR). Chest compressions were initiated, and atropine 600 µg IV was administered. These immediate interventions resulted in the return of spontaneous circulation within one minute. Electroencephalography (EEG) confirmed a deep plane of anesthesia. Upon resumption of the procedure, further manipulation of the maxilla again precipitated bradycardia, which resolved after a second 600 µg dose of atropine. No further episodes of bradycardia were noted. Following intraoperative consultation with cardiology, a postoperative 12-lead ECG was performed, which demonstrated sinus bradycardia at 53 bpm with normal morphology, as well as sinus arrhythmia.

Postoperatively, repeat 12-lead ECGs were carried out in recovery every 30 minutes until return to the ward was deemed suitable. A chest X-ray showed no evidence of pneumothorax or hemothorax, and no abnormalities apart from historic rib fractures. However, bradycardia of 44 bpm persisted until the next morning. The cardiologist indicated that follow-up Holter monitoring was not required, as this was considered a transient vagal response and not a sustained heart block. The patient was discharged the following day uneventfully (postoperative day 1) and has had an otherwise unremarkable recovery following orthognathic surgery.

## Discussion

The present case illustrates a pronounced intraoperative manifestation of TCR during maxillary mobilization, prompting a review of the existing literature to better define its clinical features, precipitating factors, and outcomes in the context of elective orthognathic surgery. This review identified a small number of reported cases, underscoring both the rarity of the phenomenon and the importance of prompt recognition and management. 

Methods

This study was undertaken in accordance with the Preferred Reporting Items for Systematic Reviews and Meta-analysis (PRISMA) guidelines.

Search strategy and selection criteria

A systematic literature search was performed using MEDLINE, PubMed Central, Embase, Scopus, and the Web of Science Core Collection on December 24, 2025, with no limitation on the timing of publication. Search terms for the reflex included “trigeminocardiac reflex,” “trigeminovagal reflex,” and “oculocardiac reflex”; terms for outcomes included “asystole,” “bradycardia,” “cardiac arrest,” and “arrhythmia”; and terms for procedures included “orthognathic” and “maxillofacial.” Full search strings are provided in Appendix 1. Studies were considered for inclusion based on the following: any case report or case series reporting on patients undergoing orthognathic surgery who demonstrated sufficient bradycardia or hemodynamic instability to allow recognition of a TCR. Cases were considered consistent with the TCR if a sudden onset of bradycardia (≥20% reduction from baseline) or asystole occurred temporally with trigeminal nerve manipulation and resolved with cessation of the stimulus or administration of an anticholinergic.

Exclusion criteria included case reports or series of the TCR in the context of non-orthognathic surgery, reports providing no data on patient outcomes, literature reviews, theoretical studies, or studies on nonhuman subjects. Recommendations and expert opinions were also excluded if they did not conform to the inclusion criteria above. Titles, abstracts, and full texts were independently screened by two authors according to the inclusion and exclusion criteria. References of identified articles were iteratively screened for additional records.

Data extraction

The following fields were extracted from each study: year of publication, patient age, patient sex, comorbidities, indication for orthognathic surgery, baseline skeletal pattern and planned movements, estimated blood loss, anesthetic agents used for induction and maintenance, duration of asystole or severity of bradycardia, precipitating factor for the TCR, management of the reflex, patient outcomes, and follow-up investigations.

Critical appraisal

The quality of included case reports was assessed using the JBI Collaboration Case Reports Critical Appraisal Tool [6]. This tool consists of eight questions assessing the quality of the case presentation, the description of diagnoses and interventions, and the quality of any takeaway lessons. The output of the tool was modified in this study to provide a numeric score of overall quality (0-8).

Study selection

**Figure 1:**
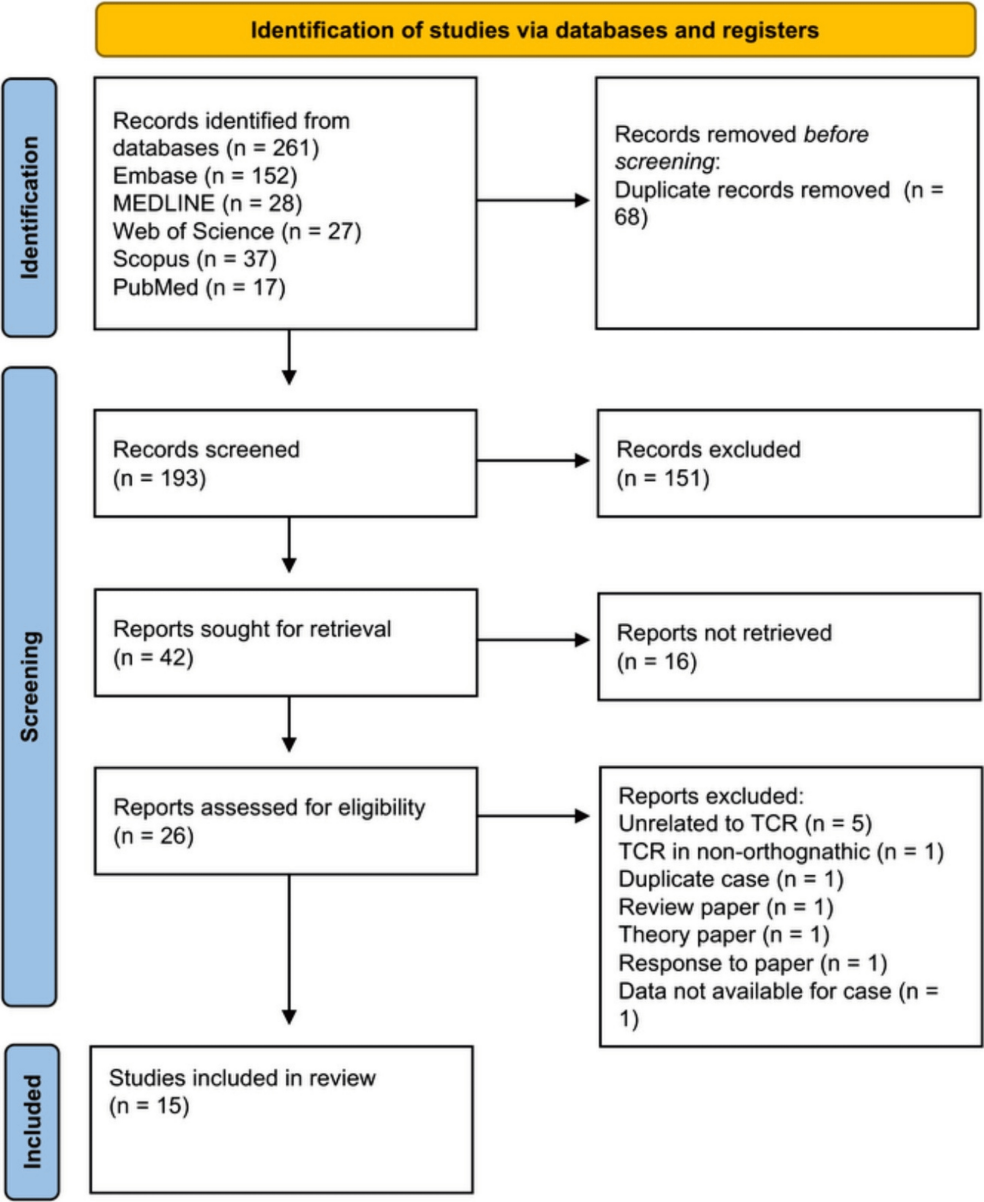
PRISMA flow diagram of study selection PRISMA: Preferred Reporting Items for Systematic Reviews and Meta-Analysis; TCR: trigeminocardiac reflex

The initial literature search identified 261 studies from five databases, 68 of which were excluded following de-duplication (Figure 1). Of these, 15 studies met the inclusion criteria. Although Precious and Skulsky (1990) met the inclusion criteria, reporting six cases of the TCR in Le Fort I osteotomy, details could not be extracted specifically for orthognathic cases; therefore, this study is not included in the analyses below, although it is described in the discussion and included in the overall case count within the review, as shown in the PRISMA flow diagram (Figure 1). Within the 14 remaining studies, 17 cases are reported, in addition to the current case.

Assessment of case report quality

The JBI Collaboration Case Reports Critical Appraisal Tool was used to assess case report quality. The median score was 8 (range 5-8), indicating that the majority of case reports provided comprehensive details of the cases included. The generally high appraisal scores supported inclusion of all identified cases, although variability in reporting quality reinforced the descriptive nature of the analysis.

Patient demographics and planned procedures

The median age of the 18 included cases was 27 years (range, 17-45 years). Nine procedures were performed in male patients; the remaining nine patients were female. With respect to the planned procedure, two cases were scheduled to undergo Le Fort I osteotomy only; four cases were planned for Le Fort I osteotomy, BSSO, and genioplasty; eight cases were planned for Le Fort I osteotomy and BSSO; two cases were planned for BSSO only; and one case was planned for Le Fort I and Hofer osteotomy (Table 2) [2,3,7-17].

**Table 2:** Characteristics and outcomes of included cases BSSO: bilateral sagittal split osteotomy; LA: local anesthetic; HR: heart rate; CPR: cardiopulmonary resuscitation; PMH; ECG: electrocardiography

Case number	Study (citation)	Age (years)/sex	Past medical history	Planned procedure	Anesthetic details	Asystole/event	Inciting event	Management of reflex	outcome
1	Aggarwal K, et al. (2020) [7]	20 M	Cleft repair aged 6 years	Le Fort I osteotomy	Not reported	Transient bradycardia 40/min	Maxillary mobilization	Mobilization discontinued; procedure recommenced more gradually following HR recovery.	Follow-up not reported
2	Alshalawi H, et al. (2024) [9]	32 M	Nil	Le Fort I osteotomy, BSSO, and genioplasty	Not reported	Transient bradycardia 25/min	Downward digital pressure on maxilla following osteotomy	Mobilization discontinued; atropine administered	Admission for 2 days for observation; postoperative ECG unremarkable. Discharged
3	Barnard NA, et al. (1990) [8]	26 F	Nil	Le Fort I osteotomy	Premedication: diazepam; induction with sodium thiopental and vecuronium; maintenance with enflurane	Transient bradycardia to 25/min with a "notable drop in blood pressure"	Maxillary downfracture	Mobilization discontinued; atropine administered	Postoperative ECG unremarkable. Discharged
4	Baronos S, et al. (2019) [10]	26 M	Not reported	Le Fort I osteotomy, BSSO, and genioplasty	Premedication midazolam; propofol, fentanyl, and rocuronium induction; desflurane and remifentanil maintenance	Asystole for 10 seconds	Insertion of bite block following completion of both Le Fort and BSSO	Glycopyrrolate administered, and procedure completed	No postoperative interventions. Discharged
5	Campbell R, et al. (1994) [17]	35 F	Nil	Le Fort I and Hofer osteotomy	Premedication midazolam; thiopental, fentanyl and succinylcholine induction; maintenance enflurane	Asystole for 10 seconds	Tuberosity osteotomy	Surgical stimulus discontinued, and enflurane vaporizer switched off	Follow-up not reported
6	Hasegawa R, et al. (2025) [13]	39 F	Nil	Le Fort I osteotomy and BSSO	Induction propofol, remifentanil, and rocuronium; propofol and remifentanil maintenance	Transient bradycardia to 46/min	Maxillary downfracture	Downfracture discontinued, and recommenced following LA infiltration	No further investigation
7	Hasegawa R, et al. (2025) [13]	26 M	Nil	Le Fort I osteotomy and BSSO	Induction propofol, remifentanil, and rocuronium. Sevoflurane, remifentanil, and fentanyl maintenance	Transient bradycardia to 52/min, with associated drop in BP to 54/25 mmHg	Mandibular split during BSSO	Pause to surgical stimulus; administered ephedrine hypochloride	No further investigation
8	Kim H (2019) [16]	23 M	Nil	BSSO	Premedication midazolam. Propofol, remifentanil, and rocuronium induction. Sevoflurane and remifentanil maintenance	Transient bradycardia to 25/min	During fixation of BSSO (post-osteotomy)	Surgery paused, and resumed following recovery of HR	Postoperative ECG unremarkable
9	Lang S, et al. (1991) [11]	28 F	Congenital ventricular septal defect	Unilateral segmental maxillary advancement	Oral clonidine premedication. Induction with thiopentone, sufentanil, and atracurium. Isoflurane for maintenance	Asystole 3 seconds	Mobilization of maxillary segment	Pause to surgery and atropine administered	Patient monitored in recovery uneventfully and discharged
10	Lang S, et al. (1991) [11]	26 F	Nil	Le Fort I osteotomy, BSSO, and genioplasty	Induction using thiopentone, fentanyl, and succinylcholine. Maintenance using isoflurane and fentanyl	Asystole; duration not reported	Subperiosteal placement of channel retractor during BSSO	Pause to surgery and lidocaine (local) and atropine administered	Postoperative ECG demonstrated sinus arrhythmia only
11	Lang S, et al. (1991) [11]	38 F	Nil	Le Fort I osteotomy and BSSO	Induction using thiopentone, alfentanil and succinylcholine. Maintenance using halothane	Transient bradycardia from 95/min baseline to 65/min	Maxillary mobilization	Pause to maxillary mobilization	Follow-up not reported
12	Maharaj K, et al. (2020) [14]	45 M	Not reported	Le Fort I osteotomy and BSSO	Not Reported	Transient bradycardia; severity not specified	Maxillary mobilization	Pause to procedure. Recommenced following recovery of HR	Follow-up not reported
13	Miyamoto J, et al. (2012) [12]	18 F	Childhood asthma aged 5 years	Le Fort I osteotomy, BSSO, and genioplasty	Induction using propofol, rocuronium and fentanyl; sevoflurane and fentanyl for maintenance	Bradycardia 12/min; presumed asystole given CPR commenced	During closure of genioplasty incision (post-completion of all osteotomies)	CPR commenced; atropine administered, and procedure completed	Postoperative ECG unremarkable. Admission for continued observation and discharged 2 weeks post-procedure
14	Ortiz-Peces L, et al. (2025) [2]	36 M	Obesity	Le Fort I osteotomy and BSSO	Induction using midazolam, fentanyl, propofol, and rocuronium. Maintenance using propofol and dexmedetomidine	Asystole 5 seconds	During dysjunction of mandible following BSSO	Pause to surgery and atropine administered	Follow-up not reported
15	Ragno JR, et al. (1989) [3]	17 M	Acne	Le Fort I osteotomy and BSSO	Diazepam pre-medication. Induction using thiopental, succinylcholine. Maintenance using isoflurane and sufentanil	Asystole; duration not reported	Maxillary downfracture	Pause to procedure. Recommenced following recovery of HR	Overnight stay on the ward. Additional investigation not reported
16	Sanuki T, et al. (2009) [15]	31 F	Nil	BSSO	Induction using propofol and rocuronium. Maintenance using sevoflurane and remifentanil	Asystole 8 seconds	Soft tissue dissection to access mandible for BSSO	Pause to surgery and atropine administered	Follow-up not reported
17	Sugiyama S, et al. (2020) [18]	31 F	Nil	Le Fort I osteotomy and BSSO	Not reported	Transient bradycardia 29/min	During dysjunction of mandible following BSSO	Pause to surgery and lidocaine (local) and atropine administered	Follow-up not reported
18	Current case	21 M	Nil	Le Fort I osteotomy and BSSO	Induction with propofol, remifentanil, and atracurium. Maintenance using propofol and remifentanil	Asystole 20 seconds	Maxillary mobilization	Pause to surgery and atropine administered; CRP commenced	Persistent bradycardia 44/min until morning following event. Discharged without complication. No cardiology follow-up recommended

Anesthetic details

All studies, with the exception of four, provided details on induction anesthesia, paralysis, and maintenance [7,9,14,18]. Among the reported studies, IV induction was used in all cases. Thiopental sodium, as expected, was utilized in older studies, whereas propofol was used in the remaining reported cases [3,8,11,17]. Maintenance anesthesia was achieved through inhalation in 11 cases, and via intravenous propofol (TIVA) in the remaining cases (including the current case) [2,3,8,10-13,15-17].

Trigeminocardiac reflex and inciting events

The severity of hemodynamic disturbance was variably reported, limiting precise comparison between cases. Transient bradycardia and asystole were reported as the manifestations of the TCR, with 10 cases reporting bradycardia, and the remaining eight (including the current case) specifically reporting asystole [2,3,7-11,13-18]. Although Miyamoto et al. (2012) did not specify “asystole,” asystole (rather than bradycardia alone) was inferred, given that the surgical team reportedly commenced cardiopulmonary resuscitation, implying a loss of cardiac output [12]. Of those presenting with asystole, duration was not specified in Lang et al. or Ragno et al. [3,11]. In studies specifying duration, asystole ranged from 3 seconds to 20 seconds in the current case [11].

The most common identified trigger was maxillary downfracture or mobilization following Le Fort I osteotomy, reported in nine (50%) cases (including the current case) [3,7-9,11,13,14]. The reflex was otherwise noted in the following instances: three cases reported the reflex during completion of dysjunction following BSSO; following maxillary tuberosity osteotomy; following insertion of a bite block (post-completion of osteotomy); following subperiosteal mandibular insertion of a channel retractor; during fixation of the BSSO; during soft tissue access to the mandible for BSSO; and during closure following completion of a Le Fort I osteotomy, BSSO, and genioplasty [2,10,11-13,15-18].

Outcome

The procedure was completed in all cases. All reports described a surgical pause during the period of either bradycardia or asystole. Five cases reported simply continuing the procedure without complication following recovery of cardiovascular parameters; nine cases (including the current case) reported administration of atropine; three reported infiltration of additional local anesthesia prior to resumption; one reported administration of glycopyrrolate; and one reported use of ephedrine [2,3,7-16,18]. Cardiopulmonary resuscitation was commenced in two cases, including the current case, with recovery to baseline cardiovascular parameters in all other cases [12].

No cases reported any significant adverse complications postoperatively. In only one case, the patient was admitted for observation and discharged after a protracted period, though the exact reasons for continued admission for two weeks postoperatively were not documented [12]. Table 3 provides a summary of patient and operative characteristics of reported cases and outcomes.

**Table 3:** Summary of patient characteristics, operative characteristics, and outcomes from identified cases ^*^Given that a number of studies reported multiple interventions in response to the TCR, percentages in the management column will equate to greater than 100%. BSSO: bilateral sagittal split osteotomy; TIVA: total intravenous anesthesia; LA: local anesthetic; TCR: trigeminocardiac reflex

Characteristic	Outcome	Frequency, n(%)
Age, Years (median(range))	27 (17-45)	-
Gender, female (n(%))	9 (50)	-
Planned procedure (n(%))	Le Fort I osteotomy	2 (11.1)
	Le Fort I, BSSO, and genioplasty	4 (22.2)
	Le Fort I and BSSO	8 (44.4)
	BSSO only	2 (11.1)
	Le Fort I, Hofer osteotomy	1 (5.6)
	Segmental advancement	1 (5.6)
Anaesthetic induction (n(%))	Thiopental sodium	4 (28.6)
	Propofol	10 (71.4)
Anaesthetic maintenance (n(%))	Inhalation	11 (78.6)
	TIVA	3 (21.4)
Event (n(%))	Bradycardia	10 (55.6)
	Asystole	8 (44.4)
Inciting event (n(%))	Maxillary downfracture/mobilization	9 (50)
	BSSO dysjunction	3 (16.7)
	Maxillary tuberosity osteotomy	1 (5.6)
	Insertion bite block	1 (5.6)
	Subperiosteal mandibular retraction	1 (5.6)
	Fixation of BSSO	1 (5.6)
	Soft tissue incision for BSSO	1 (5.6)
	Post-osteotomy closure	1 (5.6)
Duration of asystole (if present), seconds (median(range))	9 (3-20)	
Management (n(%))^*^	Pause to surgery only	5 (27.8)
	Administration of atropine	9 (50)
	Administration of LA	3 (16.7)
	Administration of glycopyrrolate	1 (5.6)
	Administration of ephedrine	1 (5.6)
	Cardiopulmonary resuscitation	2 (11.1)
Outcome (n(%))	Procedure completed	18 (100)

Discussion

**Figure 2:**
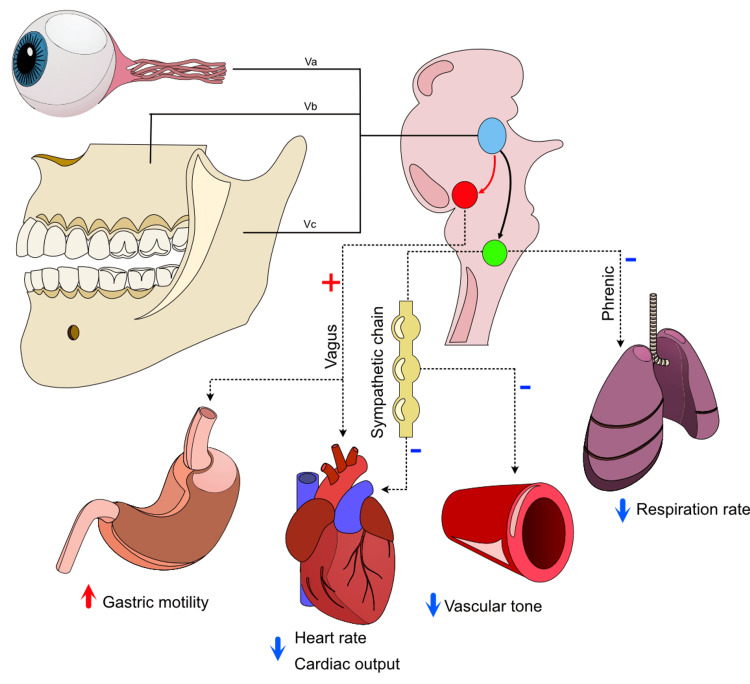
Afferent and efferent pathways of the TCRs Trigeminal afferents via the ophthalmic, maxillary, and mandibular divisions signal to the sensory nucleus of the trigeminal nerve (blue nucleus) through the Gasserian ganglion. The trigeminal sensory ganglion communicates through short internuncial fibers to the reticular formation (red arrow), and thence to the motor nucleus of the vagus nerve (red nucleus). Stimulation of the parasympathetic nerve coordinates bradycardia and gastric hypermobility. Sympathetic inhibition, via a poorly characterized mechanism (green nucleus), is thought to account for the profound drop in peripheral vascular tone and apnea observed during the reflex. TCR: trigeminocardiac reflex Image credits: Authors

The TCR is a brainstem reflex resulting from stimulation of trigeminal afferents, manifesting as bradycardia or asystole, reduction in peripheral vascular tone, and apnea. The reflex is thought to be mediated via an interaction between stimulated trigeminal afferents and both sympathetic and parasympathetic outflow at the level of the midbrain (Figure 2) [4]. The current review aimed to assess the characteristics of the TCR in orthognathic surgery.

This systematic review identified a small number of reported cases of TCR occurring during orthognathic surgery. This reflects the rarity of the phenomenon in the context of elective maxillofacial procedures. In total, 17 previously published cases were identified, in addition to the case presented in this review, spanning a wide age range and including both male and female patients undergoing a variety of procedures. Although the absolute number of reported cases is small, the reflex manifested in several instances as profound bradycardia or transient asystole, often occurring abruptly. Importantly, all reported cases, including the current case, resolved with prompt recognition and management, and no adverse long-term outcomes were documented.

The clinical manifestations ranged from transient bradycardia to complete ventricular asystole, with reported durations varying from a few seconds to twenty seconds in the present case. Despite the severity of some episodes, hemodynamic recovery was rapid following cessation of the stimulus, often supplemented by anticholinergic administration. Only two reported cases (including the current case) required cardiopulmonary resuscitation, with all procedures ultimately completed. An additional feature supporting the unpredictability and heterogeneity of this reflex is the timing during orthognathic surgery. While the majority of cases reported an obvious inciting stimulus, both Baronos et al. [10] and Miyamoto et al. [12] describe the onset of bradycardia post-completion of an uneventful osteotomy, during either closure or positioning of a bite block, suggesting that prior stimulation of the trigeminal nerve may contribute to sensitization to subsequent, relatively innocuous stimuli. Further, both in the current case and in the identified cases, there were no obvious changes in heart rate variability or physiology prior to the index event that may have been useful as a proxy to identify the likelihood of TCR occurrence.

The findings of this review are consistent with the observations reported by Precious and Skulsky in their prospective study of hemodynamic changes during orthognathic surgery [19]. In their cohort, significant bradycardia was most frequently observed during maxillary downfracture associated with Le Fort I osteotomy, with the authors proposing stimulation of maxillary branches of the trigeminal nerve as the likely mechanism. Although the term TCR was not widely used at the time, the clinical features described in the article align closely with contemporary definitions of the reflex, including abrupt onset, association with surgical manipulation, and prompt resolution following cessation of the stimulus. Importantly, Precious and Skulsky demonstrated that hemodynamic changes during maxillary mobilization were often transient and reversible, and they emphasized the effectiveness of immediate interruption of the surgical maneuver as the primary management strategy. These findings reinforce that, although largely unexpected, the TCR in orthognathic surgery is typically reversible when promptly recognized and managed appropriately.

In addition to the nature of the surgical stimulus, both the method and plane of anesthesia have been proposed as potentially contributory factors influencing the likelihood of the reflex occurring. Meuwly et al. demonstrated a strong association between lighter planes of anesthesia and increased susceptibility to the reflex, suggesting that insufficient suppression of brainstem reflex pathways may lower the threshold for activation [20]. With increasing reliance on and reliability of intraoperative depth of anesthesia monitoring, this relationship is of particular relevance to contemporary orthognathic practice. Clinical observations have suggested that propofol may exert an inhibitory influence on trigeminal reflex pathways, leading to speculation that TIVA could reduce the likelihood of the reflex compared with inhalational agents such as sevoflurane, particularly during procedures involving profound trigeminal stimulation such as a Le Fort I osteotomy, though evidence to support this observation is limited. However, cases in the present review occurred under both inhalational and intravenous techniques, and inconsistent reporting of anesthetic depth and physiological parameters precludes definitive conclusions.

The potential contribution of adjunctive anesthetic agents that modulate autonomic tone also warrants consideration. Remifentanil, a short-acting opioid widely used in orthognathic surgery, is known to increase parasympathetic activity and vagal tone. This, in effect, predisposes patients to bradycardia [20]. Clonidine, a centrally acting α2-adrenergic agonist, reduces sympathetic outflow and has been shown to enhance parasympathetic cardiac activity beyond its sedative and analgesic effects [21]. When administered in combination, remifentanil and clonidine may have a synergistic parasympathomimetic effect, potentially lowering the threshold for activation of vagally mediated reflexes such as the TCR. In the present case, the concurrent use of both agents may therefore have contributed to the severity of the observed response, although causality cannot be inferred from this single case.

Further to this, neither remifentanil nor clonidine should be regarded as independent precipitants of the reflex, and both agents are widely and safely used in anesthesia for maxillofacial procedures. Based on known autonomic physiology, rather, their combined autonomic effects may be clinically relevant in the presence of a strong trigeminal stimulus, especially when other permissive factors such as light anesthesia are present concurrently. At present, there is insufficient clinical evidence to support modification of standard anesthetic practice solely to mitigate the risk of an episode of the TCR, and indeed, causality cannot be inferred based on the identified cases presented here. Nonetheless, awareness of the potential additive vagotonic effects of these agents may assist anesthetists in anticipating and promptly managing hemodynamic instability during high-risk surgical maneuvers.

The elective nature of the discussed orthognathic surgeries and the typically young, otherwise healthy patient cohort heighten the clinical relevance of these findings. Episodes of severe bradycardia or asystole in this setting are unexpected and may cause significant intraoperative disruption if not promptly addressed. The consistent resolution of events following immediate cessation of surgical stimulation underscores the importance of close communication between surgeon and anesthetist, particularly during high-risk maneuvers such as maxillary mobilization. Anticipation and early recognition of the reflex remain central to effective management.

This review has several limitations. The available evidence is derived almost exclusively from isolated case reports or small case series, introducing publication and reporting bias. There is marked heterogeneity in terminology, diagnostic criteria, and reporting of perioperative variables, and denominator data are lacking, preventing accurate estimation of true incidence. In addition, incomplete reporting of anesthetic depth, physiological parameters, and postoperative investigations limits assessment of contributory factors. Consequently, the findings from this review should be interpreted as descriptive and hypothesis-generating rather than definitive.

Future research should focus on prospective data collection using standardized definitions of the TCR, with consistent reporting of surgical stimuli, anesthetic technique, depth of anesthesia, and autonomic responses. Such efforts would allow more accurate estimation of incidence and identification of modifiable risk factors. Until such data are available, heightened awareness, prompt recognition, and immediate interruption of the inciting stimulus remain crucial to effective management.

## Conclusions

The TCR is an uncommon but clinically significant event that can occur during orthognathic surgery, most frequently precipitated by maxillary mobilization following Le Fort I osteotomy, although less forceful stimuli may also provoke the reflex. This systematic review, together with the presented case of intraoperative asystole, demonstrates that TCR can occur in young, otherwise healthy patients and may manifest as profound bradycardia or transient asystole. Importantly, all reported cases were managed successfully with prompt recognition, cessation of the inciting stimulus, and appropriate pharmacological intervention, with no documented long-term adverse outcomes.

Despite increasing recognition of TCR within craniofacial and maxillofacial surgery, the true incidence of the reflex in orthognathic procedures remains difficult to define. The available literature is limited predominantly to case reports and small case series, with substantial heterogeneity in terminology, diagnostic thresholds, anesthetic techniques, and reporting of perioperative variables. Consequently, definitive conclusions regarding patient-specific or anesthetic risk factors cannot yet be drawn. Nevertheless, the findings of this review highlight the need for heightened awareness of TCR among surgeons and anesthetists performing orthognathic surgery. Anticipation of the reflex during recognized high-risk maneuvers, careful attention to depth of anesthesia regardless of technique, close hemodynamic monitoring, and effective communication within the operative team are essential to minimize disruption and ensure patient safety. At present, no preventive strategy has been shown to reliably eliminate the risk of TCR.

## Appendices

Search strings used for each database during literature search

MEDLINE

1. Trigeminocardiac Reflex/ 2. trigeminocardiac reflex.mp. 3. trigeminovagal reflex.mp. 4. oculocardiac reflex.mp. 5. 1 or 2 or 3 or 4 6. Asystole/ 7. Bradycardia/ 8. Heart Arrest/ 9. Arrhythmia/ 10. asystole.mp. 11. bradycardia.mp. 12. cardiac arrest.mp. 13. arrhythmia.mp. 14. 6 or 7 or 8 or 9 or 10 or 11 or 12 or 13 15. orthognathic surg*.mp. 16. orthognathic operat*.mp. 17. maxillofacial surg*.mp. 18. maxillofacial operat*.mp. 19. jaw surg*.mp. 20. jaw operat*.mp. 21. (orthognathic adj3 (surg* or operat*)).mp. 22. (maxillofacial adj3 (surg* or operat*)).mp. 23. (jaw* adj3 (surg* or operat*)).mp. 24. 15 or 16 or 17 or 18 or 19 or 20 or 21 or 22 or 23 25. 5 and 14 and 24 26. limit 25 to humans

Embase

('trigeminocardiac reflex'/exp OR trigeminocardiac reflex:ti,ab,kw OR trigeminovagal reflex:ti,ab,kw OR oculocardiac reflex:ti,ab,kw) AND ('asystole'/exp OR 'bradycardia'/exp OR 'cardiac arrest'/exp OR 'arrhythmia'/exp OR asystole:ti,ab,kw OR bradycardia:ti,ab,kw OR cardiac arrest:ti,ab,kw OR arrhythmia:ti,ab,kw) AND ((orthognathic NEAR/3 (surg* OR operat*)) OR (maxillofacial NEAR/3 (surg* OR operat*)) OR (jaw* NEAR/3 (surg* OR operat*))):ti,ab,kw AND [humans]/lim

Web of Science

#1 -TS=("trigeminocardiac reflex" OR "trigeminovagal reflex" OR "oculocardiac reflex")

#2 -TS=(asystole OR bradycardia OR "cardiac arrest" OR arrhythmia*)

#3 -TS=((orthognathic NEAR/3 (surg* OR operat*)) OR (maxillofacial NEAR/3 (surg* OR operat*)) OR (jaw* NEAR/3 (surg* OR operat*)))

#4 - #1 AND #2 AND #3

Scopus

TITLE-ABS-KEY(("trigeminocardiac reflex" OR "trigeminovagal reflex" OR "oculocardiac reflex") AND (asystole OR bradycardia OR "cardiac arrest" OR arrhythmia*) AND ((orthognathic W/3 surg*) OR (orthognathic W/3 operat*) OR (maxillofacial W/3 surg*) OR (maxillofacial W/3 operat*) OR (jaw* W/3 surg*) OR (jaw* W/3 operat*)))

Pubmed

("Trigeminocardiac Reflex"[Mesh] OR "trigeminocardiac reflex"[tiab] OR "trigeminovagal reflex"[tiab] OR "oculocardiac reflex"[tiab]) AND ("Asystole"[Mesh] OR "Bradycardia"[Mesh] OR "Heart Arrest"[Mesh] OR "Arrhythmias, Cardiac"[Mesh] OR asystole[tiab] OR bradycardia[tiab] OR "cardiac arrest"[tiab] OR arrhythmia*[tiab]) AND (orthognathic[tiab] OR maxillofacial[tiab] OR jaw[tiab]) Filters: Humans
